# Duplicated *flavonoid 3’-hydroxylase* and *flavonoid 3’, 5’-hydroxylase* genes in barley genome

**DOI:** 10.7717/peerj.6266

**Published:** 2019-01-15

**Authors:** Alexander V. Vikhorev, Ksenia V. Strygina, Elena K. Khlestkina

**Affiliations:** 1 Novosibirsk State University, Novosibirsk, Russia; 2 Institute of Cytology and Genetics, Siberian Branch of the Russian Academy of Sciences, Novosibirsk, Russia; 3 N.I. Vavilov All-Russian Research Institute of Plant Genetic Resources (VIR), St. Petersburg, Russia

**Keywords:** Flavonoid pigments, Anthocyanin biosynthesis, Gene duplication, *Hordeum*, Gene evolution, Near-isogenic lines, P450, CYP75

## Abstract

**Background:**

Anthocyanin compounds playing multiple biological functions can be synthesized in different parts of barley (*Hordeum vulgare* L.) plant. The diversity of anthocyanin molecules is related with branching the pathway to alternative ways in which dihydroflavonols may be modified either with the help of flavonoid 3′-hydroxylase (*F*3′*H*) or flavonoid 3′,5′-hydroxylase (*F*3′5′*H*)—the cytochrome P450-dependent monooxygenases. The *F3*′*H* and *F3*′5′*H* gene families are among the least studied anthocyanin biosynthesis structural genes in barley. The aim of this study was to identify and characterise duplicated copies of the *F3*′*H* and *F*3′5′*H* genes in the barley genome.

**Results:**

Four copies of the *F3*′5′*H* gene (on chromosomes 4HL, 6HL, 6HS and 7HS) and two copies of the *F3*′*H* gene (on chromosomes 1HL and 6HS) were identified in barley genome. These copies have either one or two introns. Amino acid sequences analysis demonstrated the presence of the flavonoid hydroxylase-featured conserved motifs in all copies of the *F*3′*H* and *F*3′5′*H* genes with the exception of *F*3′5′*H*-3 carrying a loss-of-function mutation in a conservative cytochrome P450 domain. It was shown that the divergence between *F3*′*H* and *F3*′5′*H genes* occurred 129 million years ago (MYA) before the emergence of monocot and dicot plant species. The *F3*′*H* copy approximately occurred 80 MYA; the appearance of *F3*′5′*H* copies occurred 8, 36 and 91 MYA. qRT-PCR analysis revealed the tissue-specific activity for some copies of the studied genes. The *F3*′*H*-1 gene was transcribed in aleurone layer, lemma and pericarp (with an increased level in the coloured pericarp), whereas the *F3*′*H-2* gene was expressed in stems only. The *F3*′5′*H-1* gene was expressed only in the aleurone layer, and in a coloured aleurone its expression was 30-fold higher. The transcriptional activity of *F3*′5′*H-2* was detected in different tissues with significantly higher level in uncoloured genotype in contrast to coloured ones. The *F3*′5′*H-3* gene expressed neither in stems nor in aleurone layer, lemma and pericarp. The *F3*′5′*H-4* gene copy was weakly expressed in all tissues analysed.

**Conclusion:**

*F3*′*H* and *F3*′5′*H*-coding genes involved in anthocyanin synthesis in *H. vulgare* were identified and characterised, from which the copies designated *F3*′*H-1*, *F3*′*H-2*, *F3*′5′*H-1* and *F3*′5′*H-2* demonstrated tissue-specific expression patterns. Information on these modulators of the anthocyanin biosynthesis pathway can be used in future for manipulation with synthesis of diverse anthocyanin compounds in different parts of barley plant. Finding both the copies with tissue-specific expression and a copy undergoing pseudogenization demonstrated rapid evolutionary events tightly related with functional specialization of the duplicated members of the cytochrome P450-dependent monooxygenases gene families.

## Introduction

Plant phenolic compounds flavonoids and their coloured derivatives anthocyanins are secondary metabolites providing important functions (Grotewold, 2006a; Grotewold, 2006b). Flavonoids are ubiquitously present in plant cells. They are involved in the regulation of developmental processes, in the protection against biotic and abiotic stress and in the attraction of seed dispersers and pollinators (Khlestkina, 2013; Pourcel et al., 2007; Landi, Tattini & Gould, 2015). Due to their antioxidant activity, these compounds are also useful for the health of plant foods consumers—humans and animals (Khoo et al., 2017; Chaves-Silva et al., 2018).

**Figure 1:**
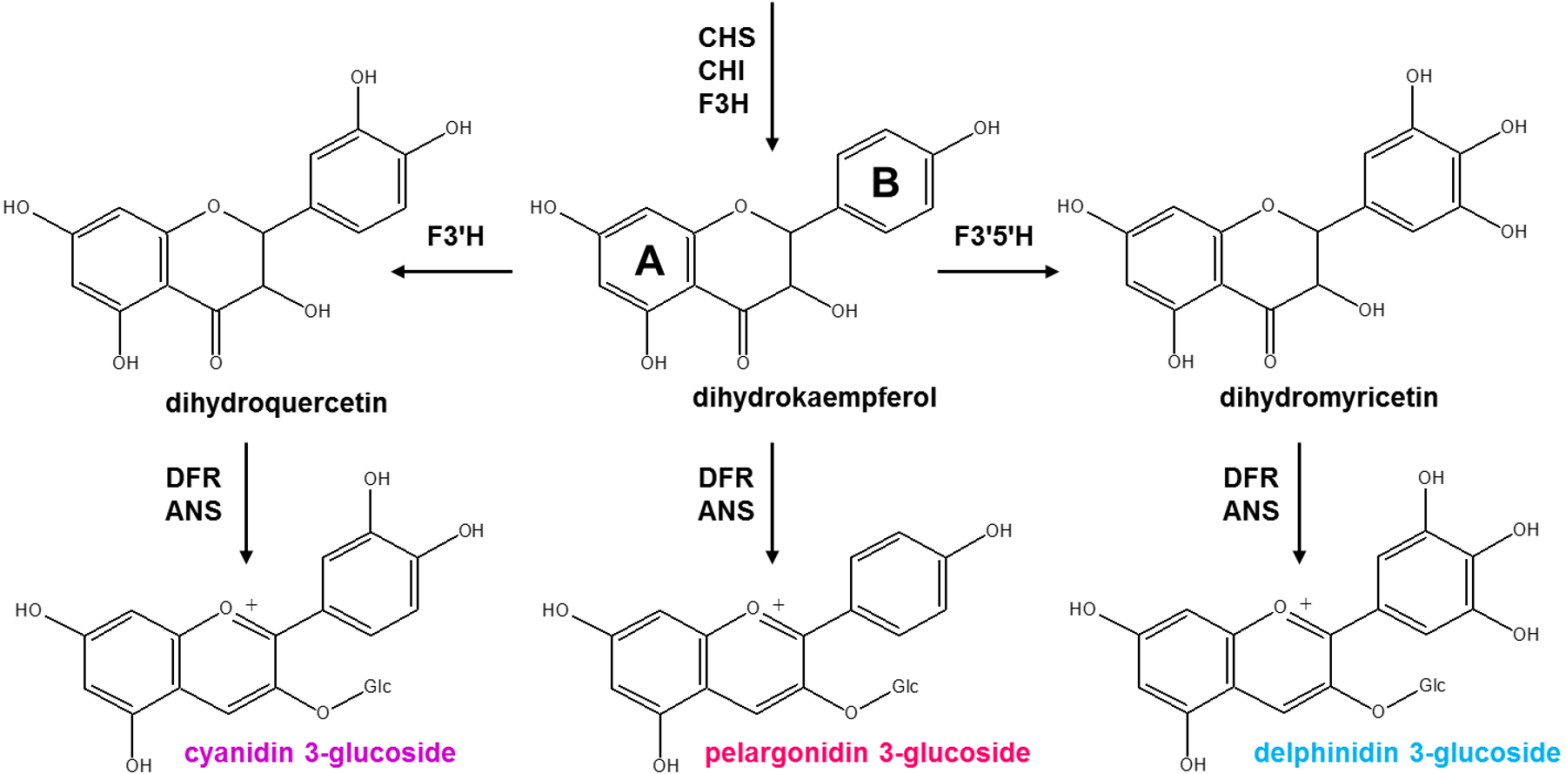
Scheme of anthocyanidins biosynthesis. The enzymes are indicated in red: ANS, anthocyanidin synthase; CHI, chalcone-flavanone isomerase; CHS, chalcone synthase; DFR, dihydroflavonol 4-reductase; *F*3*H*, flavanone 3-hydroxylase; *F*3′*H*, flavonoid 3′-hydroxylase; *F*3′5′*H*, flavonoid 3′, 5′-hydroxylase.

Cytochrome P450 (also called CYP) proteins, named for the absorption band at 450 nm, are one of the largest proteins superfamilies (Werck-Reichhart & Feyereisen, 2000). These proteins are found in all organisms from protists to mammals, but their number has exploded in plants. Flavonoid 3′-hydroxylase (*F*3′*H*, CYP75B, EC 1.14.13.21) and flavonoid 3′, 5′-hydroxylase (*F*3′5′*H*, CYP75A, EC 1.14.13.88) are cytochrome P450-dependent monooxygenases that require NADPH as a co-factor (Tanaka & Brugliera, 2013). These enzymes are involved in the biosynthesis of anthocyanin compounds—glycosylated forms of anthocyanidins producing by the flavonoid biosynthesis pathway (Fig. 1). *F*3′*H* and *F*3′5′*H* compete for substrate recruitment and hydroxylate 3′ or 3′5′ position of dihydroflavonols for the parallel synthesis of delphinidin and cyanidin, the precursors of blue and reddish-purple pigments (Tanaka, Brugliera & Chandler, 2009; Tanaka & Brugliera, 2013). Barley (*Hordeum vulgare* L.) is an important agricultural crop. In addition to the photosynthetic pigments giving a green colour, barley produces pigments that form diverse colouration patterns of different parts of plant. Purple and blue anthocyanins are accumulated in barley grains in the pericarp and aleurone layer, respectively (Adzhieva et al., 2015; Shoeva, Strygina & Khlestkina, 2018). Despite the fact that the genes coding the enzymes involved in anthocyanin biosynthetic pathway is well understood at the genetic and molecular level, the least studied genes in this branch are *F3*′*H* and *F3*′5′*H*. Because of useful properties of anthocyanin compounds, the study of genes involved in the anthocyanin biosynthesis is important. Previously, the presence of one *F3*′*H* gene copy (*F3′H-1*) expressing in genotype with purple pericarp was shown, as well as the presence of one *F3*′5′*H* copy (*F3′5′H-1*) with aleurone specific expression (Shoeva et al., 2016; Strygina, Börner & Khlestkina, 2017). Since the fact of tissue-specific activity of these genes and the fact that these anthocyanin compounds can be accumulated in other parts of the plant, it was concluded that there should be other copies of the *F3′H* and *F3′5′H* genes. The aim of this study was the identification and characterization of the *F3′H* and *F3′5′H* genes copies in the barley genome with Bowman’s near-isogenic lines (NILs) contrasting in anthocyanin pigmentation: ‘BW’ (Bowman), ‘PLP’ (purple lemma and pericarp) and ‘BA’ (intense blue aleurone).

## Materials & Methods

### Identification and structural analysis of duplicated genes

The homologous nucleotide sequences of *F3′H-1* (GenBank: AK362052) and *F3′5′H-1* (GenBank: MF679159) were found in databases IPK Barley BLAST Server (https://webblast.ipk-gatersleben.de/barley_ibsc/), BARLEX (http://apex.ipk-gatersleben.de/apex/f?p=284:10) and EnsemblPlants (http://plants.ensembl.org/index.html) using BLASTN search (*p*-value = 0.001). The multiple sequence alignment was made using Multalin (http://multalin.toulouse.inra.fr/multalin/). The gene structure was predicted using FGENESH+ software (http://www.softberry.com/berry.phtml?topic=fgenes_plus&group=programs&subgroup=gfs) using predicted polypeptide sequences of *F3′H-1* and *F3′5′H-1*. The available promoter sequences were analysed with New PLACE database (https://sogo.dna.affrc.go.jp/cgi-bin/sogo.cgi?lang=en&pj=640&action=page&page=newplace). The annotation of the functional domains was carried out using InterPro: protein sequence analysis & classification (https://www.ebi.ac.uk/interpro/). Modelling of the tertiary structure of the predicted amino acid sequences was performed using SWISS-MODEL (https://swissmodel.expasy.org/). Using MEGA v6.06 software (http://www.megasoftware.net) with 1,000 bootstrap replicates to assess the branch support the construction of the UPGMA tree, the calculation of *Ka*/*Ks* ratio and calculation of divergence time was performed. The calibration of timeclock was based on divergence time between barley and maize (50–60 MYA) (Salse et al., 2009; Cheng et al., 2012; Subburaj et al., 2016) and potato and petunia (30 MYA) (Kamenetzky et al., 2010).

### Plant material, RNA extraction, cDNA synthesis

Plant material exploited for gene expression analysis included the cultivar Bowman of barley *H. vulgare* and two Bowman’s near-isogenic lines (NILs): ‘BW’ (Bowman, NGB22812), ‘PLP’ (purple lemma and pericarp, NGB22213) and ‘BA’ (intense blue aleurone, NGB20651). The set of the lines was provided by the Nordic Gene Bank (NGB, http://www.nordgen.org). PLP NIL have reddish-purple pericarp and stems due to the presence of *PLP* loci (chromosomes 2AL and 7HS); BA NIL have blue aleurone layer to the presence of *BA* loci (chromosomes 4HL and 7HL). The plants for RNA extraction from aleurone layers, pericarps, lemmas and stems were grown in ICG Greenhouse Core Facilities (Novosibirsk, Russia) under a 12 h photoperiod at 20–25 °C. The experiments were conducted in three replicates for each genotype. Aleurones and pericarps were cut out with a scalpel from grains at early dough stage maturity. RNAs from aleurone layers, pericarps, lemmas and stems (collected at the end of flowering) were extracted using a RNeasy Mini Kit (QIAGEN, Hilden, Germany) followed by DNase treatment with RNase-free DNase set (QIAGEN, Hilden, Germany). To obtain single-stranded cDNA samples total RNA was converted in a 20-µL reaction mixture from a template consisting of 0.4 µg of total RNA using a RevertAid First Strand cDNA Synthesis Kit (Thermo Fisher Scientific Inc., Waltham, MA, USA).

**Table 1:** Gene-specific primers used for qPCR analysis of structural *F*3′*H* and *F*3′5′*H* genes of barley.

Gene	Forward primer (5′→3′)	Reverse primer (5′→3′)	PCR product length (bp)
*F3′H-1*	GCCAGGGAGTTCAAGGACA	CTCGCTGATGAATCCGTCCA	168
*F3′H-2*	AGGATAATCGCCCAGAGAAGG	GCCATCGCCCACTCCAC	203
*F3′5′H-1*	ATCGCATGTCGTGGCTATG	GCCGAGTTCACCATCATTTC	143
*F3′5′H-2*	CACAGACCTCAACATCAAAGC	TCCATCTCCGCCTGTGCT	138
*F3′5′H-3*	GAACGGCGTCACAGACAT	TCCATCTCCGCCTGTGCT	148
*F3′5′H-4*	GAACGACGACGGCGAGAC	CGCCATTGCCCACTCCAC	109

### Primer design and qRT-PCR

Gene-specific primer pairs were constructed using Oligo Primer Analysis Software v.7 (https://www.oligo.net/) based on sequences found in IPK Barley BLAST Server (Table 1). The qRT-PCR was based on a SYNTOL SYBR Green I kit (Syntol, Moscow, Russia). The amplifications were performed in an ABI Prism 7,000 Sequence Detection System (Applied Biosystems, http://www.lifetechnologies.com). PCR was performed in a 15-µL reaction mixture under the following conditions: 1 cycle −15 min at 95 °C; 40 cycles −15 s at 94 °C, 30 s at 60 °C, 30 s at 72 °C. The construction of PCR product melting curves under the conditions: 15 s at 95 °C; 15 s at 60 °C; 15 s at 95 °C. The reference sequence was *Ubiquitin* gene; primers were suggested in (Himi & Noda, 2005). The raw data is in File S1. Each sample was run in three technical replications. The differences among the lines were tested by Mann–Whitney *U*-test (*p* ≤ 0.05).

## Results

### Identification of F3′Hs and F3′5′Hs in barley genome

Amid all identified highly homologous protein-coding sequences to *F3′H-1* and *F3′5′H-1* genes with 85.5–97.1% identity in functionally significant domains in the barley genome, only six encode proteins belonging to the CYP75 protein class (File S2). Among the revealed genes, one CYP75B-like copy of the *F3′H-1* gene sequence (1HL; GenBank: AK362052) located on chromosome 6HS was found (File S2). The gene was designated *F3′H-2*. Its predicted full coding nucleotide sequence shares 69.6% identity with *F3′H-1*. Three CYP75A gene sequences were identified using *F3′5′H-1* gene sequence (4HL; GenBank: MF679160): two highly homologous gene copies designated *F3′5′H-2* (6HL) and *F3′5′H-3* (6HS) with a level of identity 82.6% and 83.0%, respectively, and one copy designated *F3′5′H-4* with 63.0% identity (7HS).

### Study of the structural organisation of the *F*3′*H* and *F*3′5′*H* genes

**Figure 2:**
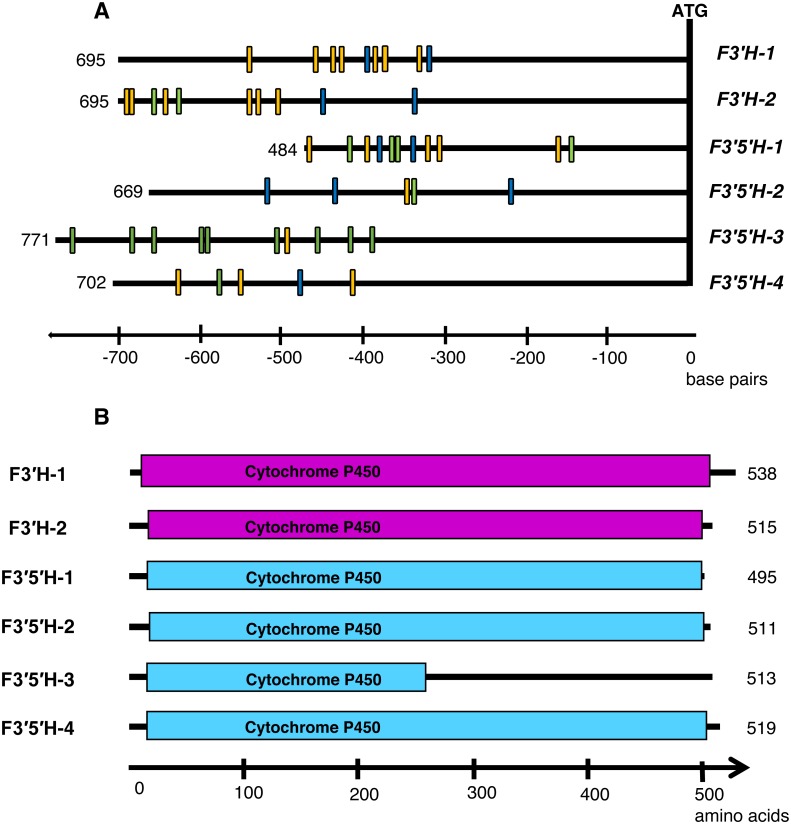
(A) Diverse promoter structure of *F3′H* and *F3′5′H* genes in barley; (B) structure analysis of identified genes. Promoter analysis was performed using New PLACE database. Orange, light-dependent motifs, blue, bHLH-type transcription factors binding elements, green, MYB-type transcription factors binding elements. Analysed protein motif is Cytochrome P450, E-class, group I (IPR002401).

All *F3′H* and *F3′5′H* genes identified in the current study in *H. vulgare* genome consist of two exons with the exception of *F3′5′H-1* having three exons. Analysis of the promoter elements for the annotated genes (∼600 bp upstream to the ATG start site) revealed many motives responsible for light-dependent activation (especially in *F3′H-1* and *F3′5′H-1*), as well as Myb-dependent and Myc-dependent elements required for genes involved in the biosynthesis of flavonoid compounds (Fig. 2A, File S3). Unlike other copies, *F3′5′H-2* and *F3′5′H-3* have only one light-induced promoter element (GATA-box).

**Figure 3:**
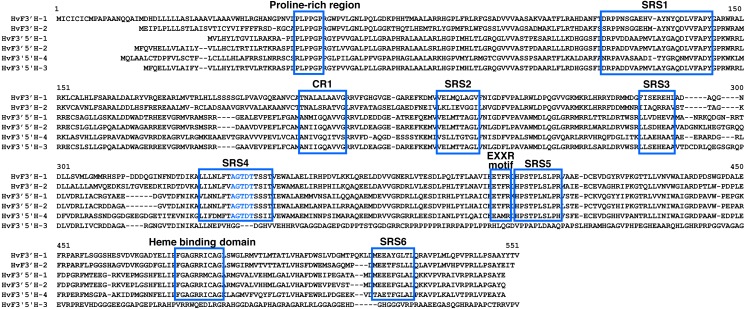
The conserved domains of the *F*3′*H* and *F*3′5′*H* protein sequences in barley. Critical motifs are indicated with blue rectangles. Blue letters, oxygen binding motif. CR1, hydroxylation activity site. SRS, substrate recognition site.

Amino acid sequences alignment with framing functional domains are shown in Fig. 2B. All the identified genes have a Cytochrome P450 domain (E-class, group I; IPR002401), however, *F3′5′H-3* gene copy carries a frameshift indel mutation, which results in the truncation of the functional Cytochrome P450 domain in the middle and affects the tertiary protein structure (Fig. 2B, File S4). These sequences also possessed the conserved domains of flavonoid hydroxylase, including proline-rich region, heme binding domain, oxygen binding motif, hydroxylation activity site (CR1), EXXR motif and substrate recognition sites (SRS) (Fig. 3). Six functional SRSs, that are important for the determination of substrate specificity in CYP75 proteins, were determined in the predicted amino acid sequences of barley *F*3′*Hs* and *F*3′5′*Hs*. In *F*3′5′*H-3* only three SRS, proline-rich and CR1 motifs are present (Fig. 3). All other barley CYP75s have not lost their functional domains.

### Evolutionary analysis of CYP75 genes

The number of non-synonymous substitutions per non-synonymous sites (*Ka*), the number of synonymous substitutions per synonymous sites (*Ks*) and the *Ka*/*Ks* ratio for *CYP75* genes of barley were calculated. Synonymous and non-synonymous substitution rates ranged between 0.541–0.685 and 0.269–0.461 for identified paralogs, respectively (File S5). Using the formula *Ka*/*Ks*, it was predicted that *F3′H* and *F3′5′H* paralogs may be under stabilising selection (*Ka*/*Ks* is close to 0.5) with the exception of *F3′5′H-3*. This copy may experience neutral selection since the *Ka*/*Ks*_*F*3′5′*H*__-3_ is close to one (File S5).

**Figure 4:**
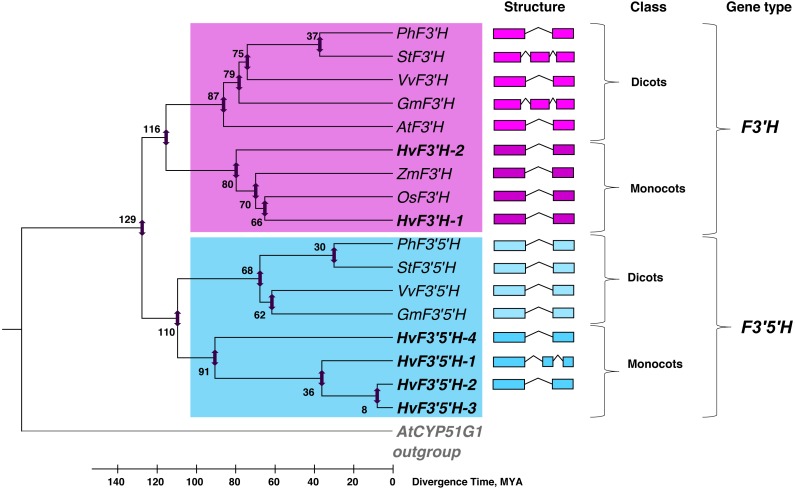
The analysis of phylogenetic similarity of the *F3′H* and *F3′5′H* genes based on full coding sequences. The phylogenetic tree reconstruction and the estimation of divergence time were made in MEGA 6.06. The UPGMA method with 1,000 bootstrap replicates was used for construction of phylogenic tree. The structure of genes is shown. Blue cluster, genes coding *F3′5′H*, pink cluster, genes coding *F3′H* in monocot and dicot plant species. *AtCYP51G1*, NM_101040; *AtF3′H*, NM_120881; *AtF3′5′H*, NM_120881; *PhF3′H*, AF155332; *PhF3′5′H*, Z22544; *VvF3′H*, NM_001280987; *VvF3′5′H*, NM_001281235; *OsF3′H*, XM_015757555; *ZmF3′H*, CM000781; *StF3′H*, XM_006345070; *StF3′5′H*, NM_001287878; *GmF3′H*, NM_001250086; *GmF3′5′H*, NM_001249703.

The phylogeny of *F3′H* and *F3′5′H* genes was analysed using complete coding sequences of identified genes from genome of barley and other angiosperm species. The phylogenetic tree indicated that *F3′H* and *F3′5′H* families form two separate clusters (Fig. 4, blue and purple clusters, respectively); within each one clearly divided into two groups—monocot and dicot plant species. It was assumed that *F3′H* and *F3′5′H* genes are the results of duplication and neofunctionalization of the single *CYP75* gene in a genome of the common ancestor of monocot and dicot plant species. The analysis of genetic similarity and the divergence time calculation revealed that this event occurred about 129 million years ago (MYA) (Fig. 4) shortly before the monocots and dicots divergence (estimated time is 110–116 MYA).

In addition, we calculated the time of segmented duplications in *H. vulgare* genome with the formation of paralogous gene copies (Fig. 4). The *F*3′*H* copy apparently occurred before the divergence of Triticeae tribe from rice and maize about 80 MYA. In contrast, the *F*3′5′*H* in barley genome was duplicated at least three times: 91, 36 and 8 MYA. Thus, the last formation of the *F*3′5′*H* copy occurred after the separation of *Hordeum* genera from the common *Triticeae* ancestor (approximately 9–11 MYA Cheng et al., 2012; Subburaj et al., 2016).

### Analysis of the *F*3′*H* and *F*3′5′*H* genes expression

**Figure 5:**
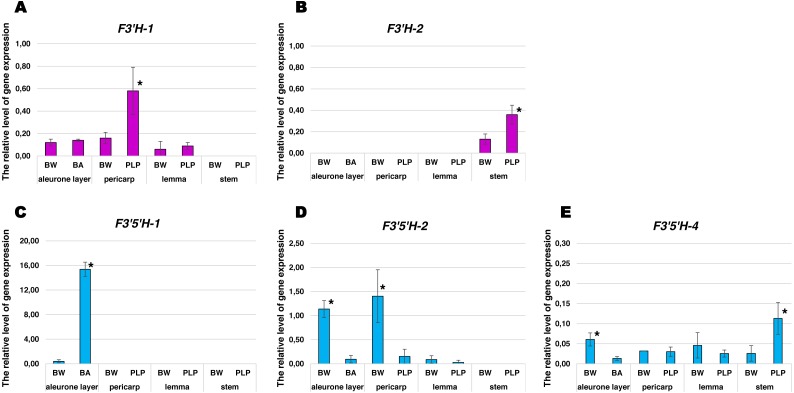
Expression of *F3′H* and *F3′5′H* genes in barley cv Bowman and Bowman NILs contrasting for anthocyanin pigmentation. (A) *F3′H-1*. (B) *F3′H-2*. (C) *F3′5′H-1*. (D) *F3′5′H-2*. (E) *F3′5′H-4*. Barley tissues: aleurone layer, pericarp, lemma and stems. The data is presented as mean value ± standard error. An asterisk (*) indicates a statistically significant difference between Bowman and its NIL, with *p* ≤ 0.05 (Mann–Whitney *U*-test).

Comparative analysis of relative gene expression levels was performed using RNAs isolated from the aleurone layer, pericarp, lemma and stems of the Bowman’s near-isogenic lines (NILs) contrasting in anthocyanin pigmentation: ‘BW’ (Bowman, NGB22812), ‘PLP’ (purple lemma and pericarp, NGB22213) and ‘BA’ (intense blue aleurone, NGB20651) (File S6). It was found, that the *F3′H-1* gene was expressed in aleurone layer, pericarp and lemma with an increased expression level in a pigmented pericarp of ‘PLP’ (3.6 times higher than in uncoloured one) (Fig. 5). A tissue-specific expression was detected for the *F3′H-2* gene. Activation of the expression of this gene occurs in stems only. Moreover, in coloured stems of ’PLP’ the relative expression level was three times higher than in uncoloured stems of ‘BW’ (Fig. 5). Expression of the *F3′5′H-1* gene only in aleurone layer was confirmed (Fig. 5). It was shown that in pigmented aleurone of ‘BA’ this gene was expressed 30 times actively than in uncoloured aleurone of ‘BW’. *F3′5′H-2* was strongly expressed in pericarp and aleurone layer of ‘BW’ in comparison to coloured ones (9.3 and 12.7 times higher, respectively) (Fig. 5). Expression of the *F3′5′H-3* gene was not detected in analysed tissues. The gene *F3*′5′*H-4* was weakly expressed in all studied tissues with slight expression increasing in the pigmented stems (Fig. 5).

## Discussion

Gene duplication is an important evolutionary mechanism providing a source of genetic material for the specialization or the new gene function appearance through the mutations and selection (Proulx, 2011; Magadum et al., 2013). Evolution by gene duplication has arisen as a general principle of biological evolution, which is apparent from the prevalence of duplicated genes in all genomes of sequenced organisms (Ohno, 1970). Gene copies have occurred as a result of segmental duplications (duplication of individual genomic regions) or polyploidization (whole genome duplications) (Ohno, 1970; Lynch et al., 2001; Eichler & Sankoff, 2003).

Gene duplicates can expect one of the possible fates: pseudogenization (PG), subfunctionalization (SF) or neofunctionalization (NF) (Ohno, 1970). In the PG process, one of the gene copies loses its function after degenerate mutation acquiring, for example, in the promoter region. The NF process proposes that one gene copy retains the ancestral function while the other gets a novel function. The SF is a major process of divergence with differential division of ancestral gene functions (Ohno, 1970).

In plants, the pattern of the SF leading to tissue-specific expression is frequent. For instance, regulatory genes coding bHLH/Myc-type transcription factors controlling the anthocyanins biosynthesis in barley grain divide their functions: *HvMyc1* gene (located in 2HL chromosome) regulates accumulation of anthocyanin pigments in pericarp while *HvMyc2* gene (located in 4HL chromosome) provides the biosynthesis of anthocyanin pigments in aleurone layer like in PLP and BA NILs, respectively (Jende-Strid, 1993; Cockram et al., 2010; Strygina, Börner & Khlestkina, 2017). As an example of tissue-specification of structural anthocyanin biosynthesis genes *flavanone 3-hydroxylase* (*F3H*) genes in *Triticum aestivum* genome could be considered: the copy designated *TaF3H-B2* is transcribed specifically in roots of bread wheat while the *TaF3H-B1* gene copy is not expressed in roots but it is expressed in other different parts of the plant (Khlestkina et al., 2013).

In the flavonoid biosynthesis pathway, *F*3′*H* and *F*3′5′*H* are important enzymes controlling the hydroxylation at the 3′ and 5′ of reddish-purple and blue pigments, respectively (Tanaka, Brugliera & Chandler, 2009; Tanaka & Brugliera, 2013). In most plants, *F3*′*H* and *F3*′5′*H* genes are present in low-copy number. In the current work, we have identified duplicated copies of *F3′H* and *F3′5′H* genes in *H. vulgare* genome. We have shown that the divergence between *F3′H* and *F3′5′H* genes from the common ancestor’s *CYP75* gene occurred 129 MYA, which occurred based on our calculations 13–19 MYA years earlier than the appearance of monocot and dicot plant species (110–116 MYA according to our calculations; 90–165 MYA according to different estimates Chaw et al., 2004; Herron et al., 2009; Cheng et al., 2012). The duplication of *F3′H* and *F3′5′H* in barley genome took place several times: the *F3′H* copy arose approximately 80 MYA while the appearance of *F3′5′H* copies occurred 8, 36 and 91 MYA (Fig. 4). Thus, the first acts of duplication of both genes occurred before the origin of the family Poaceae (Gramineae) (Kellogg, 2001).

The ratio of non-synonymous (*Ka*) to synonymous (*Ks*) substitutions is used to determine the direction of natural selection after duplication: *Ka*/*Ks* > 1 implies positive selection, *Ka*/*Ks* < 1 means stabilising selection, *Ka*/*Ks* = 1 indicates neutral selection (Kondrashov et al., 2002). Analysis of duplicated *F3′H* and *F3′5′H* genes indicated that most of the identified gene copies are under stabilising selection. The exception is *F3′5′H-3* gene copy, which is supposed to be a pseudogene due to the mutation in the coding part of the gene, which breaks the reading frame and changes the protein structure. In addition, we did not detect its transcriptional activity in analysed tissues.

The genes encoding *F3′H* showed a precise tissue-specific activity likewise *TaF3H* genes of bread wheat: *F3′H-1* is expressed in aleurone layer, pericarp and lemma, while *F3′H-2* is transcriptionally active in stems only (Fig. 5). Besides, increasing of the expression level were observed in tissues with reddish-purple pigmentation (pericarp and stems) apparently provided by cyanidin derivatives (these identifications are putative due to the absence of a biochemical study of the gene products). The increase of relative expression level of *F3′H-1* in the aleurone layer or lemma was not detected in BA and PLP NILs (Fig. 5). In these tissues, there are almost or completely no cyanidin derivatives, which is evident from the phenotype of these lines (File S6). An increase in the level of gene expression in anthocyanin-pigmented plant tissues is a common feature of genes in anthocyanins biosynthesis pathway in cereals (Shoeva et al., 2015; Shoeva et al., 2016; Shoeva & Khlestkina, 2015). For example, in the pericarp of purple-grained PLP line the expression level of flavonoid biosynthesis structural genes (*CHS*, *CHI*, *F3H*, *F3*′*H*, *DFR*, *ANS*) was significantly higher than in the uncoloured Bowman, that led to total anthocyain content increase in PLP line identified by ultra-performance liquid chromatography (HPLC) (Shoeva et al., 2016).

Among the *F3′5′H* copies, only two have a tissue-specific activity: *F3′5′H-1* and *F3′5′H-2*. The *F3′5′H-1* copy was expressed only in the aleurone layer, and the level of its activity was much higher in the blue aleurone compared to the uncoloured one (Fig. 5). Aleurone-specific expression of this gene was noted earlier, and it was shown that *F3′5′H-1* is one of the key regulators of the aleurone layer pigmentation (Strygina, Börner & Khlestkina, 2017). The copy designated *F3′5′H-2* was expressed only in the barley grain. Moreover, the expression of this gene is much higher in the aleurone layer and pericarp in the green BW line in comparison to coloured ones (Fig. 5). The *F3′5′H-4* gene copy was expressed in all tissues analysed. Since there are almost no light-dependent elements in the promoters of *F3′5′H-2* and *F3′5′H-4*, it can be assumed that these gene copies encode for different isoenzymes specialised in the synthesis of such flavonoid compounds as catechin available in the barley at the high level (McMurrough, Loughrey & Hennigan, 1983; Madigan, McMurrough & Smyth, 1994). Alike specialization was demonstrated earlier for such organisms as tea plant and its relatives (Punyasiri et al., 2004; Jin et al., 2017). These results suggest the SF and diversification of *F3′Hs* and *F3′5′Hs* in the barley genome.

## Conclusions

*F*3′*H* and *F*3′5′*H*-coding genes involved in anthocyanin synthesis in *Hordeum vulgare* L. were identified and characterised. One *F3′H* (*F3′H-2*) and three *F3′5′Hs* (*F3′5′H-2*, *F3′5′H-3*, *F3′5′H-4*) were described for the first time. The subfunctionalization expressing in tissue-specific activity of *F3′H-1*, *F′H-2*, *F3′5′H-1* and *F3′5′H-2* genes was shown. It was also found that the *F3′5′H-3* gene, carrying frame-shift indel mutation, is the pseudogenic duplicate. Finding both the copies with tissue-specific expression and the *F3′5′H-3* copy undergoing pseudogenization demonstrated rapid evolutionary events tightly related with functional specialization of the duplicated members of the cytochrome P450-dependent monooxygenases gene families. The results obtained are important for understanding of the features of flavonoid biosynthesis regulation in barley.

##  Supplemental Information

10.7717/peerj.6266/supp-1 File S1 The raw data of expression of *F3′H* and *F3′5′H* genes in barley cv Bowman and Bowman NILs contrasting for anthocyanin pigmentation

10.7717/peerj.6266/supp-2 File S2 The cytochrome P450-dependent monooxygenase genes of barley identified in the current study in BARLEX and EnsemblPlants databases

10.7717/peerj.6266/supp-3 File S3 Putative *cis*-acting regulatory elements identified in the *F3′H* and *F3′5′H* promoters. Promoter analysis was performed using New PLACE database. “+” –coding strand, “–” –template strand

10.7717/peerj.6266/supp-4 File S4 Predicted three dimensional structures of *F*3′*H* and *F*3′5′*H* of barley determined using the SWISS-MODEL program

10.7717/peerj.6266/supp-5 File S5 The estimated number of non-synonymous substitutions per non-synonymous sites (*Ka*), the number of synonymous substitutions per synonymous sites (*Ks*) and the *Ka*/*Ks* ratio for barley *F3′H* and *F3′5′H* genes

10.7717/peerj.6266/supp-6 File S6 Phenotypic characteristics of Bowman’s NIL contrasting in anthocyanin pigmentation

## Additional Information and Declarations

### Data Availability

The following information was supplied regarding data availability: Raw data is available in the Supplemental Materials.

## References

[ref-1] Adzhieva VF, Babak OG, Shoeva OY, Kilchevsky AV, Khlestkina EK (2015). Molecular-genetic mechanisms underlying fruit and seed coloration in plants. Vavilovskii Zhurnal Genetiki i Selektsii (Vavilov Journal of Genetics and Breeding).

[ref-2] Chaves-Silva S, Dos Santos AL, Chalfun-Jr A, Zhao J, Peres LE, Benedito VA (2018). Understanding the genetic regulation of anthocyanin biosynthesis in plants—tools for breeding purple varieties of fruits and vegetables. Phytochemistry.

[ref-3] Chaw SM, Chang CC, Chen HL, Li WH (2004). Dating the monocot–dicot divergence and the origin of core eudicots using whole chloroplast genomes. Journal of Molecular Evolution.

[ref-4] Cheng J, Khan MA, Qiu WM, Li J, Zhou H, Zhang Q, Gou W, Zhu T, Peng J, Sun F, Li S, Korban SS, Han Y (2012). Diversification of genes encoding granule-bound starch synthase in monocots and dicots is marked by multiple genome-wide duplication events. PLOS ONE.

[ref-5] Cockram J, White J, Zuluaga DL, Smith D, Comadran J, Macaulay M, Luo Z, Kearsey MJ, Werner P, Harrap D, Tapsell C, Liu H, Hedley PE, Stein N, Schulte D, Steuernagel B, Marshall DF, Thomas WTB, Ramsay L, Mackay I, Balding DJ, Waugh R, O’Sullivan DM, AGOUEB Consortium (2010). Genome-wide association mapping to candidate polymorphism resolution in the unsequenced barley genome. Proceedings of the National Academy of Sciences of the United States of America.

[ref-6] Eichler EE, Sankoff D (2003). Structural dynamics of eukaryotic chromosome evolution. Science.

[ref-7] Grotewold E (2006a). The genetics and biochemistry of floral pigments. Annual Review of Plant Biology.

[ref-8] Grotewold E (2006b). The science of flavonoids.

[ref-9] Herron MD, Hackett JD, Aylward FO, Michod RE (2009). Triassic origin and early radiation of multicellular volvocine algae. Proceedings of the National Academy of Sciences of the United States of America.

[ref-10] Himi E, Noda K (2005). Red grain color gene (R) of wheat is a Myb-type transcription factor. Euphytica.

[ref-11] Jende-Strid B (1993). Genetic control of flavonoid biosynthesis in barley. Hereditas.

[ref-12] Jin JQ, Ma JQ, Yao MZ, Ma CL, Chen L (2017). Functional natural allelic variants of flavonoid 3′, 5′-hydroxylase gene governing catechin traits in tea plant and its relatives. Planta.

[ref-13] Kamenetzky L, Asís R, Bassi S, De Godoy F, Bermudez L, Fernie AR, Wan P, Vrebalov J, Giovannoni JJ, Rossi M, Carrari F (2010). Genomic analysis of wild tomato introgressions determining metabolism-and yield-associated traits. Plant Physiology.

[ref-14] Kellogg EA (2001). Evolutionary history of the grasses. Plant Physiology.

[ref-15] Khlestkina E (2013). The adaptive role of flavonoids: emphasis on cereals. Cereal Research Communications.

[ref-16] Khlestkina EK, Dobrovolskaya OB, Leonova IN, Salina EA (2013). Diversification of the duplicated F3h genes in Triticeae. Journal of Molecular Evolution.

[ref-17] Khoo HE, Azlan A, Tang ST, Lim SM (2017). Anthocyanidins and anthocyanins: colored pigments as food, pharmaceutical ingredients, and the potential health benefits. Food & Nutrition Research.

[ref-18] Kondrashov F, Rogozin I, Wolf Y, Koonin E (2002). Selection in the evolution of gene duplications. Genome Biology.

[ref-19] Landi M, Tattini M, Gould KS (2015). Multiple functional roles of anthocyanins in plant-environment interactions. Environmental and Experimental Botany.

[ref-20] Lynch M, O’Hely M, Walsh B, Force A (2001). The probability of preservation of a newly arisen gene duplicate. Genetics.

[ref-21] Madigan D, McMurrough I, Smyth MR (1994). Determination of proanthocyanidins and catechins in beer and barley by high-performance liquid chromatography with dual-electrode electrochemical detection. Analyst.

[ref-22] Magadum S, Banerjee U, Murugan P, Gangapur D, Ravikesavan R (2013). Gene duplication as a major force in evolution. Journal of Genetics.

[ref-23] McMurrough I, Loughrey MJ, Hennigan GP (1983). Content of (+)-catechin and proanthocyanidins in barley and malt grain. Journal of the Science of Food and Agriculture.

[ref-24] Ohno S (1970). Evolution by gene duplication.

[ref-25] Pourcel L, Routaboul J-M, Cheynier V, Lepiniec L, Debeaujon I (2007). Flavonoid oxidation in plants: from biochemical properties to physiological functions. Trends in Plant Science.

[ref-26] Proulx SR (2011). Multiple routes to subfunctionalization and gene duplicate specialization. Genetics.

[ref-27] Punyasiri PAN, Abeysinghe ISB, Kumar V, Treutter D, Duy D, Gosch C, Martens S, Forkmann G, Fischer TC (2004). Flavonoid biosynthesis in the tea plant Camellia sinensis: properties of enzymes of the prominent epicatechin and catechin pathways. Archives of Biochemistry and Biophysics.

[ref-28] Salse J, Abrouk M, Bolot S, Guilhot N, Courcelle E, Faraut T, Waugh R, Close TJ, Messing J, Feuillet C (2009). Reconstruction of monocotelydoneous proto-chromosomes reveals faster evolution in plants than in animals. Proceedings of the National Academy of Sciences of the United States of America.

[ref-29] Shoeva OY, Khlestkina EK (2015). The specific features of anthocyanin biosynthesis regulation in wheat. Advances in wheat genetics: from genome to field.

[ref-30] Shoeva OY, Kukoeva TV, Börner A, Khlestkina EK (2015). Barley Ant1 is a homolog of maize C1 and its product is part of the regulatory machinery governing anthocyanin synthesis in the leaf sheath. Plant Breeding.

[ref-31] Shoeva OY, Mock H-P, Kukoeva TV, Börner A, Khlestkina EK (2016). Regulation of the flavonoid biosynthesis pathway genes in purple and black grains of Hordeum vulgare. PLOS ONE.

[ref-32] Shoeva OY, Strygina KV, Khlestkina EK (2018). Genes determining the synthesis of flavonoid and melanin pigments in barley. Vavilovskii Zhurnal Genetiki i Selektsii (Vavilov Journal of Genetics and Breeding).

[ref-33] Strygina KV, Börner A, Khlestkina EK (2017). Identification and characterization of regulatory network components for anthocyanin synthesis in barley aleurone. BMC Plant Biology.

[ref-34] Subburaj S, Cao S, Xia X, He Z (2016). Phylogenetic analysis, lineage-specific expansion and functional divergence of seed dormancy 4-like genes in plants. PLOS ONE.

[ref-35] Tanaka Y, Brugliera F (2013). Flower color and cytochromes P450. Philosophical Transactions of The Royal Society B.

[ref-36] Tanaka Y, Brugliera F, Chandler S (2009). Recent progress of flower color modification by biotechnology. International Journal of Molecular Sciences.

[ref-37] Werck-Reichhart D, Feyereisen R (2000). Cytochromes P450: a success story. Genome Biology.

