# Supplementary material for: Duplicated flavonoid 3’-hydroxylase and flavonoid 3’, 5’-hydroxylase genes in barley genome
Source: PeerJ. 2019 Jan 15;7:e6266. doi: 10.7717/peerj.6266 (PMC6338099; doi:10.7717/peerj.6266)
Supplement: File S2 [file peerj-07-6266-s002.docx]

**Additional file 2.**

The cytochrome P450-dependent monooxygenase genes of barley identified in the current study.

| **№** | **Location**  **(EnsemblPlant)** | **Gene hit** | **Subject**  **(IPK)** | **Percentage, %**  ***F*3*′H-1* / *F3′5′H-1*** | **Annotation**  **(BARLEX)** | **Gene** |
| --- | --- | --- | --- | --- | --- | --- |
| 1. | Chromosome chr1H: 556,691,612-556,693,908 | HORVU1Hr1G094880 | morex_contig_1575828; | 100 / 69 | Cytochrome P450 flavonoid 3',5'-hydroxylase | *F3′H-1* |
| 2. | Chromosome chr4H: 534,190,658-534,192,411 | HORVU4Hr1G063780 | morex_contig_1575914 | 71 / 99 | Cytochrome P450 superfamily protein | *F3′5′H-1* |
| 3. | Chromosome chr6H: 6,328,401-6,331,022 | HORVU6Hr1G002400 | morex_contig_1570242; | 73 / 63 | Cytochrome P450 flavonoid 3',5'-hydroxylase | *F3′H-2* |
| 4. | Chromosome chr6H: 563,621,087-563,622,955 | HORVU6Hr1G087250 | morex_contig_1635985;  morex_contig_139393; | 76 / 80  68 / 81 | Cytochrome P450 superfamily protein | *F3′5′H-2* |
| 5. | Chromosome chr6H: 563,810,378-563,812,260 | HORVU6Hr1G087310 | morex_contig_366097; | 68 / 82 | Flavonoid 3',5'-hydroxylase | *F3′5′H-3* |
| 6. | Chromosome chr7H: 9,879,963-9,884,227 | HORVU7Hr1G007580 | morex_contig_241646;  morex_contig_1582601; | 67 / 67  - / 70 | Cytochrome P450 superfamily protein | *F3′5′H-4* |
