# Supplementary material for: Duplicated flavonoid 3’-hydroxylase and flavonoid 3’, 5’-hydroxylase genes in barley genome
Source: PeerJ. 2019 Jan 15;7:e6266. doi: 10.7717/peerj.6266 (PMC6338099; doi:10.7717/peerj.6266)
Supplement: File S3 [file peerj-07-6266-s003.docx]

**Additional file 3.**

Putative cis-acting regulatory elements identified in the *F3'H* and *F3'5'H* promoters. Promoter analysis was performed using New PLACE database.

“+” – coding strand, “–” – template strand.

| Motif | Sequence | Gene | Position (chain orientation) | | Description |
| --- | --- | --- | --- | --- | --- |
|  |  |  | “+” | “- ‘’ |  |
| PALBOXAPC | CCGTCC | *F3'5'H-1* | - | - 480 | One of three putative cis- acting elements (boxes P, A, and L) of phenylalanine ammonia- lyase (PAL; EC 4.3.1.5) genes in parsley. These elements appear to be necessary but not sufficient for elicitor- or light- mediated PAL gene activation. |
|  |  | *F3'5'H-4* | - 631 | - |  |
| SORLIP1AT | GCCAC | *F3'H-1* | - 467  -  - | -  - 358  - 367 | One of "Sequences Over- Represented in Light- Induced Promoters (SORLIPs) in Arabidopsis. |
|  |  | *F3'5'H-1* | - 328 | - |  |
| SORLIP2AT | GGGCC | *F3'5'H-1* | - 396 |  | One of "Sequences Over- Represented in Light- Induced Promoters (SORLIPs) in Arabidopsis. |
| SORLIP5AT | GAGTGAG | *F3'H- 1* | -  -  - 376 | - 442  - 438  - | One of "Sequences Over- Represented in Light- Induced Promoters (SORLIPs) in Arabidopsis. |
| GATABOX | GATA | *F3'H- 2* | - 536 | - | Required for high level, light regulated, and tissue specific expression. |
|  |  | *F3'5'H- 1* | - 323  - | -  -160 |  |
|  |  | *F3'5'H- 2* |  | - 356 |  |
|  |  | *F3'5'H- 3* | - | - 492 |  |
|  |  | *F3'5'H- 4* | - 557 | - |  |
| IBOXCORE | GATAA | *F3'H- 2* | - | - 678 | Conserved sequence upstream of light- regulated genes of both monocots and dicots. |
|  |  | *F3'5'H- 4* | - 557 | - |  |
| GT1CONSENSUS | GRWAAW | *F3'H- 1* | - | - 545 | Consensus GT-1 binding site in many light- regulated genes. |
|  |  | *F3'H- 2* | -  -  -  - | - 679  - 649  - 525  - 505 |  |
| MYCCONSENSUSAT | CANNTG | *F3'H- 1* | - 390  - | -  - 318 | MYC recognition site found in the promoters of the dehydration- responsive gene rd22 and many other genes in Arabidopsis. |
|  |  | *F3'H- 2* | -  - 340 | - 451  - |  |
|  |  | *F3'5’H- 1* | - 382  - 356 | -  - |  |
|  |  | *F3'5'H- 2* | - 515  -  - 227 | -  -  - |  |
| MYCATERD1 | CATGTG | *F3'5'H- 1* | - | - 382 | MYC recognition sequence necessary for expression of erd1 (early responsive to dehydration) in dehydrated Arabidopsis. |
|  |  | *F3'5'H- 2* | - | - 425 |  |
| MYCATRD22 | CACATG | *F3'5'H- 1* | - 382 | - | Binding site for MYC (rd22BP1) in Arabidopsis (A.t.) dehydration- resposive gene, rd22. |
|  |  | *F3'5'H- 2* | - 425 | - |  |
| MYBZM | CCWACC | *F3'5'H- 1* | - 374 | - | Core of consensus maize P (myb homolog) binding site. |
|  |  | *F3'5'H- 3* | - | - 756 |  |
| MYBCORE | CNGTTR | *F3'5'H- 1* | - 147 | - | Binding site for all animal MYB and at least two plant MYB proteins ATMYB1 and ATMYB2. |
|  |  | *F3'5'H- 3* | - 757  -  - 667  -  - 596  - 505  - 456  - 414  - 390 | -  - 686  -  - 599  -  -  -  -  - |  |
| MYBPLANT | MACCWAMC | *F3'5'H- 1* | - 376 | - | Plant MYB binding site; Consensus sequence related to box P in promoters of phenylpropanoid biosynthetic genes such as PAL, CHS, CHI, DFR, CL, Bz1. |
|  |  | *F3'5'H- 3* | - | - 756 |  |
| MYB1AT | WAACCA | *F3'H- 2* | - 658  - | -  - 630 | MYB recognition site found in the promoters of the dehydration- responsive gene rd22 and many other genes in Arabidopsis. |
|  |  | *F3'5'H- 1* | - | - 419 |  |
|  |  | *F3'5'H- 4* | - | - 582 |  |
| MYBST1 | GGATA | *F3'5'H- 2* | - | - 356 | Core motif of MybSt1 (a potato MYB homolog) binding site. |
| MYB2CONSENSUSAT | YAACKG | *F3'5'H- 3* | -  - 599 | - 667  - | MYB recognition site found in the promoters of the dehydration- responsive gene rd22 and many other genes in Arabidopsis. |
