# Supplementary material for: Duplicated flavonoid 3’-hydroxylase and flavonoid 3’, 5’-hydroxylase genes in barley genome
Source: PeerJ. 2019 Jan 15;7:e6266. doi: 10.7717/peerj.6266 (PMC6338099; doi:10.7717/peerj.6266)

Additional file 4. Predicter three dimensional structures of F3'H and F3'5'H of barley determined using the SWISS-MODEL program.

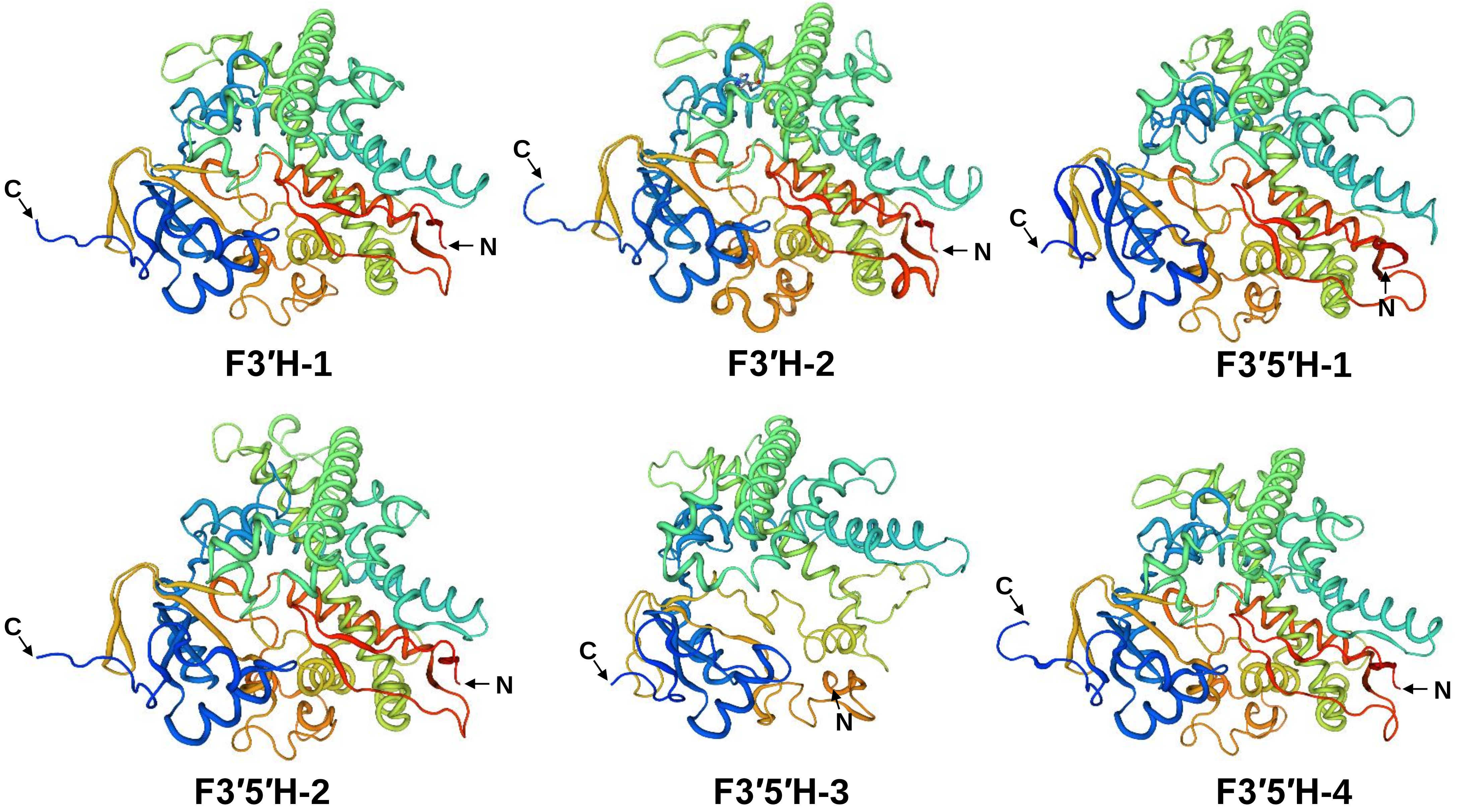

Supplement: File S4 [file peerj-07-6266-s004.pdf]
