# Supplementary material for: Duplicated flavonoid 3’-hydroxylase and flavonoid 3’, 5’-hydroxylase genes in barley genome
Source: PeerJ. 2019 Jan 15;7:e6266. doi: 10.7717/peerj.6266 (PMC6338099; doi:10.7717/peerj.6266)

Additional file 5.

The estimated number of non-synonymous substitutions per non-synonymous sites (*Ka*), the number of synonymous substitutions per synonymous sites (*Ks*) and the *Ka* / *Ks* ratio for barley *F3'H* and *F3'5'H* genes.

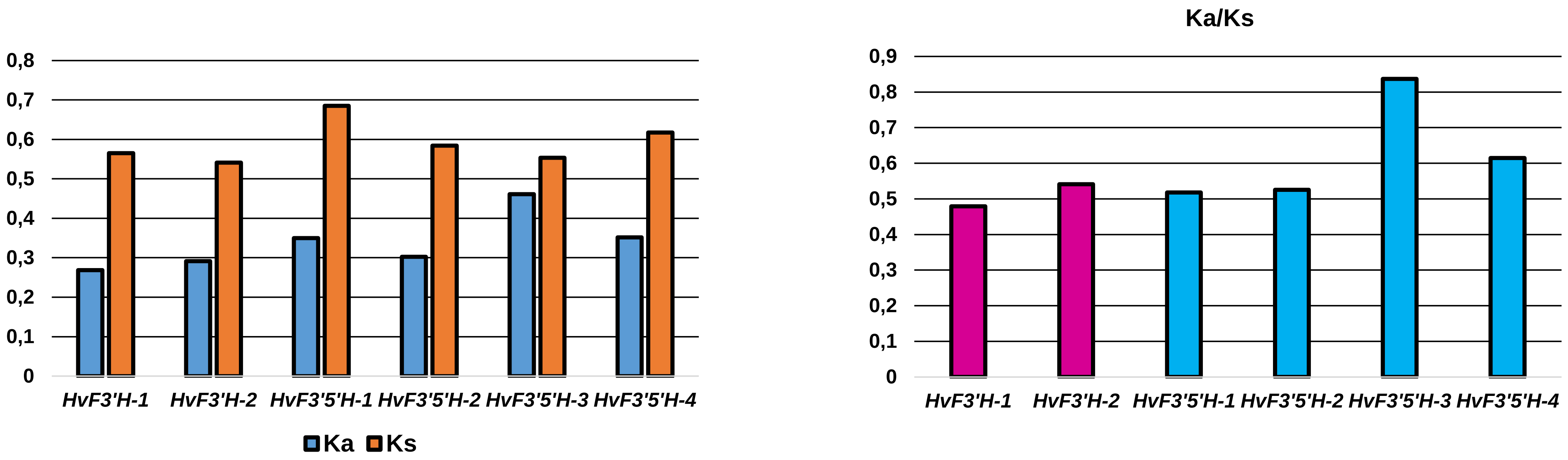

Supplement: File S5 [file peerj-07-6266-s005.pdf]
