# Supplementary material for: Duplicated flavonoid 3’-hydroxylase and flavonoid 3’, 5’-hydroxylase genes in barley genome
Source: PeerJ. 2019 Jan 15;7:e6266. doi: 10.7717/peerj.6266 (PMC6338099; doi:10.7717/peerj.6266)
Supplement: File S6 [file peerj-07-6266-s006.pdf]

Additional file 6.

Phenotypic characteristics of Bowman’s NIL contrasting in anthocyanin pigmentation.

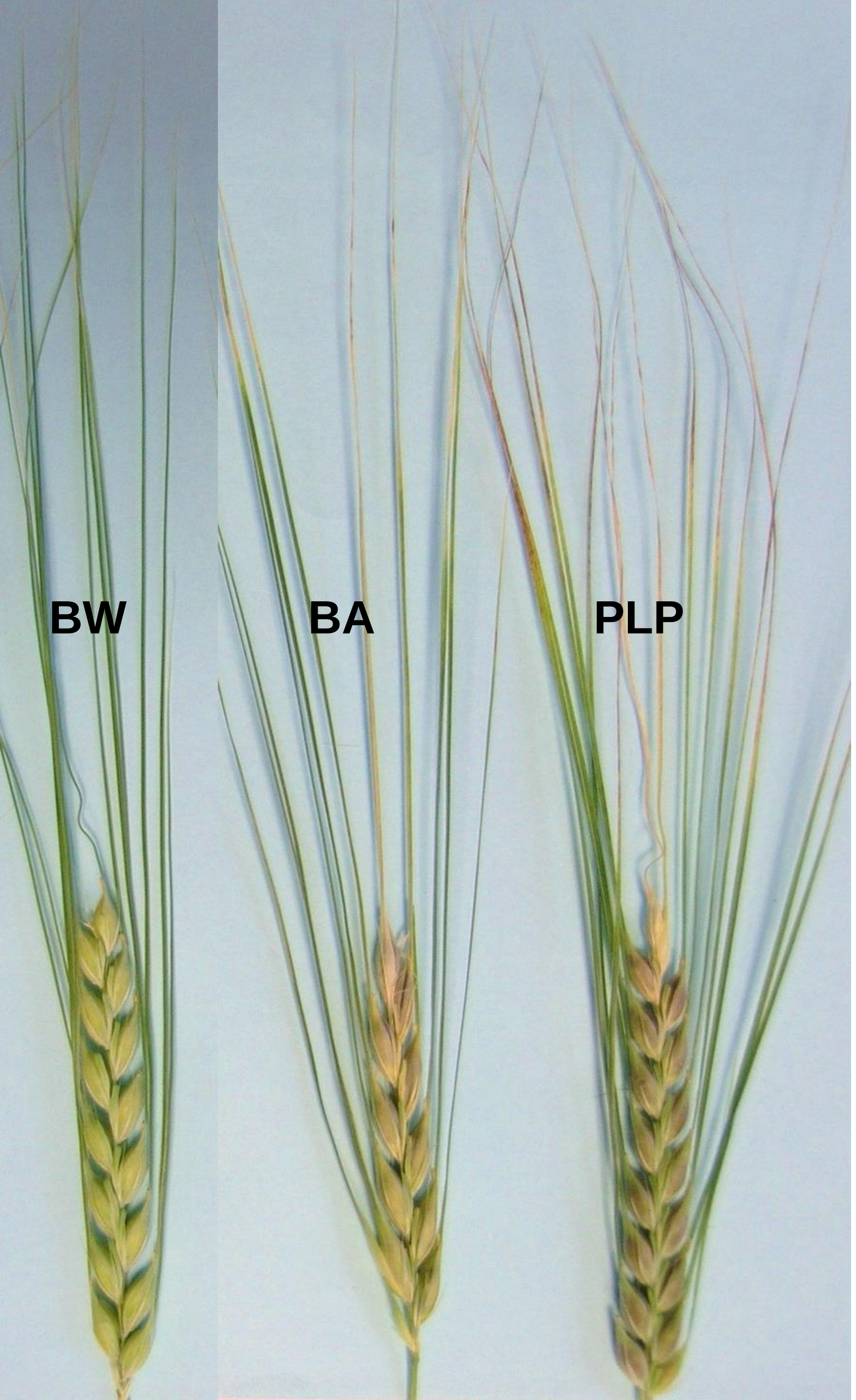

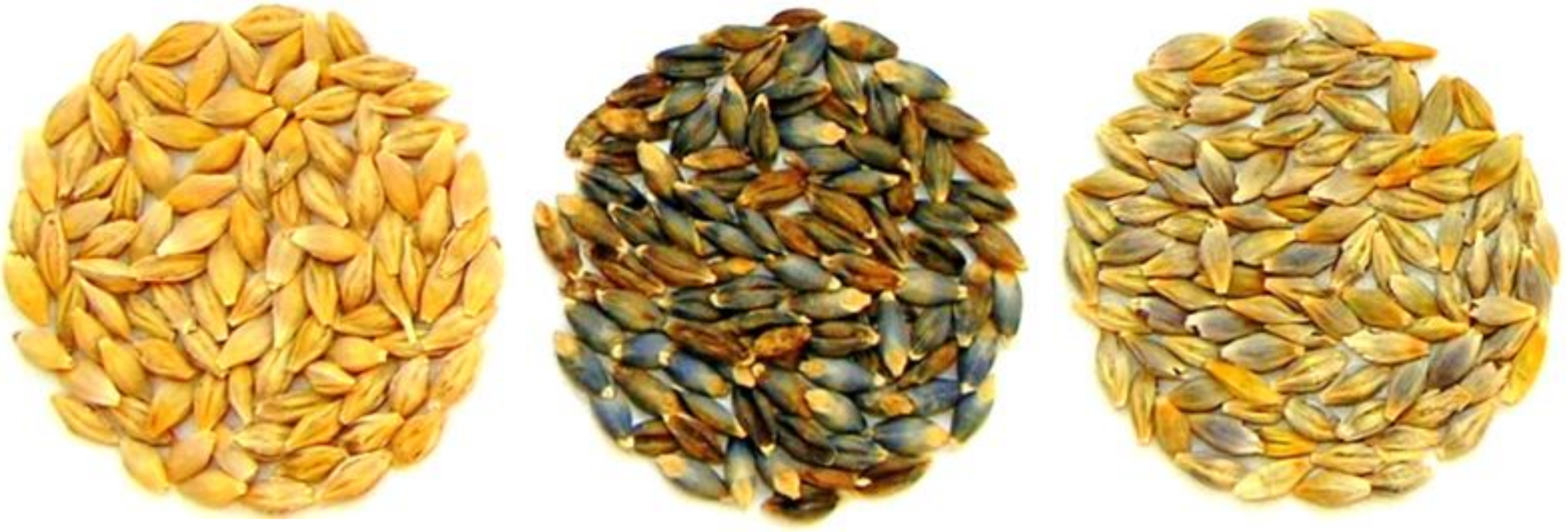

BW BA PLP

Line designation	NGB ID	Type	Anthocyanin pigmentation			
			aleurone	pericarp	lemma	stem
BW (Bowman)	NGB22812	hulled	-	-	-	-
BA (Intense blue aleurone)	NGB20651	hulless	blue	-	-	purple
PLP (Purple lemma and pericarp)	NGB22213	hulled	-	purple	-	purple
